# Identifying and Classifying Enhancers by Dinucleotide-Based Auto-Cross Covariance and Attention-Based Bi-LSTM

**DOI:** 10.1155/2022/7518779

**Published:** 2022-04-05

**Authors:** Shulin Zhao, Qingfeng Pan, Quan Zou, Ying Ju, Lei Shi, Xi Su

**Affiliations:** ^1^Institute of Fundamental and Frontier Sciences, University of Electronic Science and Technology of China, Chengdu, China; ^2^Yangtze Delta Region Institute (Quzhou), University of Electronic Science and Technology of China, Quzhou, Zhejiang, China; ^3^General Hospital of Heilongjiang Province Land Reclamation Bureau, Harbin, China; ^4^School of Informatics, Xiamen University, Xiamen, China; ^5^Department of Spine Surgery, Changzheng Hospital, Naval Medical University, Shanghai, China; ^6^Foshan Maternal and Child Health Hospital, Foshan, Guangdong, China

## Abstract

Enhancers are a class of noncoding DNA elements located near structural genes. In recent years, their identification and classification have been the focus of research in the field of bioinformatics. However, due to their high free scattering and position variability, although the performance of the prediction model has been continuously improved, there is still a lot of room for progress. In this paper, density-based spatial clustering of applications with noise (DBSCAN) was used to screen the physicochemical properties of dinucleotides to extract dinucleotide-based auto-cross covariance (DACC) features; then, the features are reduced by feature selection Python toolkit MRMD 2.0. The reduced features are input into the random forest to identify enhancers. The enhancer classification model was built by word2vec and attention-based Bi-LSTM. Finally, the accuracies of our enhancer identification and classification models were 77.25% and 73.50%, respectively, and the Matthews' correlation coefficients (MCCs) were 0.5470 and 0.4881, respectively, which were better than the performance of most predictors.

## 1. Introduction

Enhancers are short noncoding fragments of DNA sequences that can greatly enhance the activity of promoters [1]. After Benerji discovered the first 140 bp enhancer in SV40DNA in 1981, researchers attempted to find more enhancers on a genome-wide scale [2]. Among these attempts, some computer methods have been used to identify and classify the enhancers [3, 4]. For example, Jia and He extracted features using high-dimensional eigenvectors based on double-contour Bayes, nucleotide composition, and pseudonucleotide composition, realizing the distinction between enhancers and nonenhancers and strong and weak enhancers through a support vector machine (SVM) and developing a web server named EnhancerPred [5]. iEnhancer-2L [6] selected a feature extraction method, namely, pseudo *K* tuple nucleotide composition (PseKNC), and predicted them with SVM. iEnhancer-EL [7] adopted three feature extraction methods, namely, *k*-mers, subsequence profile, and PseKNC, and utilized SVM as an individual classifier for ensemble learning prediction. The Enhancer-5step [8] applied the word-embedded representation to biological sequences, specifically by using the FastText tool to extract the 100-dimensional features and then using the supervisory method SVM for predictive classification. Tan et al. [9] took six types of dinucleotide physical and chemical properties as input characteristics and employed a deep recursive neural network-based classifier integration model, which achieved good results. iEnhancer-ECNN [10] exploited convolutional neural network (CNN) integration, combined with one-hot coding and *k*-mers descriptors as sequence coding projects, and is an effective computing strategy. iEnhancer-CNN [11] extracted the features of enhancers from the original DNA sequence using word2vec and predicted them using CNN. These models and predictors continuously improve the performance of enhancer identification and classification, but the performance is not good enough in general, and further research is needed, especially the classification of enhancers.

**Figure 1:**
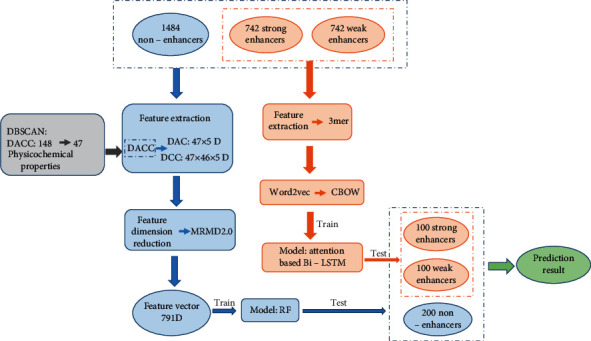
The main flow chart of the research process in this paper.

In this paper, we propose a new model building strategy; the process is shown in Figure 1. First, we divided the task into the identification and classification of enhancers. In enhancer identification, we used the density-based spatial clustering of applications with noise (DBSCAN) [12] algorithm to cluster the physicochemical properties of the original 148 dinucleotides and extract 47 of them, as detailed in Supplementary Materials (available here). Then, 11,045  (47 × 47 × 5) dimensional features were obtained by the dinucleotide-based auto-cross covariance (DACC) [13] feature extraction method. To prevent overfitting, the dimension was reduced to 791 using MRMD2.0 [14], a Python toolkit that combines seven commonly used feature ranking algorithms with the PageRank strategy. After CNN, RNN, etc., failed to achieve ideal results, the use of random forest achieved good results. In the final independent test, an accuracy of 77.5% and MCC of 0.552 were achieved. In the process of enhancer classification, we used 3-mers to split sequences and CBOW as word embedding models to transform biological sequences into 198 × 200 dimension word sequences. Then, we used attention-based bidirectional long short-term memory (Bi-LSTM) [15] to carry out predictive classification, and in independent tests, the accuracy was 65%, and the MCC was 0.3824.

Finally, we give a general introduction to the structure and organization of this work. In Results, we compared and discussed the prediction performance achieved by the enhancer identification and classification models proposed in this paper with existing models or predictors, and summarize the paper. Then, in Discussion, we introduced our models in detail and discussed the dimensionality reduction and dimension selection experiment in enhancer identification and the word2vec model parameter selection experiment in enhancer classification. Finally, in Material and Methods, the datasets, DACC feature extraction algorithm, the selection rules of physicochemical properties using DBSCAN algorithm, the principle of attention-based Bi-LSTM, and the model evaluation metrics are described, respectively.

## 2. Results

In this study, we proposed different models for enhancer identification and enhancer classification. In enhancer identification, the physicochemical properties of dinucleotides obtained by clustering screening were used for DACC feature extraction, and then, we performed feature dimension reduction. Finally, random forest was used for prediction. In enhancer classification, we used 3-mers and CBOW models to obtain word vectors and then used attention-based Bi-LSTM for classification. The model proposed in this paper finally achieved excellent performance in the independent test. Specifically, the model had 77.25%, 77.30%, 77.20%, and 0.5470 values for enhancer identification, accuracy, sensitivity, specificity, and MCC, respectively. For the enhancer classification, the performances were 73.50%, 87.00%, 60.00%, and 0.4881, respectively.  Table 1 gives a detailed comparison of the performance of the model presented in this paper and the previous models. In terms of enhancer identification, we are slightly inferior to Enhancer-5Step and iEnhancer-CNN but superior to other models. Although the performance is not absolutely excellent, we hope that the construction idea of the model has some inspiration to others. In the enhancer classification, the MCC of the model presented in this paper was significantly higher than the MCC of other models, with an increase of 0.1201 compared with the highest MCC of 0.3680, and its sensitivity was also the highest, reaching 87.00%. Both models have achieved preeminent performance.

The contribution of this paper is to use the DBSCAN clustering algorithm to select representative physical and chemical properties, and then extracted DACC features, which avoids overfitting to a certain extent. And we experimentally compared the effects of word2vec model parameters and different types of LSTM on performance. The ideas of model construction can also be applied to other bioinformatics datasets or computational biology directions [16–22] such as enhancer-promoter interaction identification [23, 24], disease biomarker mining [25–31], and drug discovery [32–34].

In the future research, we will try to optimize the DBSCAN algorithm in terms of adaptive selection of parameters to improve its processing of different density datasets. And deep learning can indeed achieve better results than ordinary machine learning algorithms in enhancer classification. We will try hot deep learning technologies such as graph neural networks to further improve prediction performance.

## 3. Discussion

### 3.1. Enhancer Identification

Feature extraction is a vital link in building an excellent classification model. In this paper, to obtain DACC feature vectors, we use iLearn [35] to extract them. A total of 148 dinucleotide physicochemical properties were provided by iLearn [35]. If the DACC in the form of all physicochemical properties is adopted, a total of 109,520 dimensions of features will be obtained, but the sample size is relatively small, and overfitting is easily generated. Therefore, in this study, our solution was to use DBSCAN to conduct cluster screening for physical and chemical property indexes.

DBSCAN is a commonly used density-based clustering method. Compared with *K*-means, the DBSCAN algorithm does not need to predefine the number of clusters and DBSCAN can find clusters of arbitrary shapes. In addition, DBSCAN can also identify “outliers”, and the “outliers” are the special physical and chemical properties we want to find. At present, many studies have improved DBSCAN to enable it to process large datasets at a high speed.

In this paper, clustering and processing of physicochemical dinucleotide indexes are carried out. After the treatment, we obtained 47 kinds of physical and chemical property indexes. Then, feature extraction was carried out through DACC. After executing the iLearn [35] command line, 47 × 47 × 5 (11,045) feature dimensions were obtained: python iLearn-nucleotide-acc.py --file data. txt --method DACC --type DNA --lag 5.

Considering that there are still more features in 11,045 dimensions, we tried to use MRMD2.0 [36–38] for feature dimension reduction. MRMD2.0 integrates rich feature selection algorithms and feature ranking algorithms and is superior to the single feature selection algorithm. We conducted dimension reduction three times, and the fivefold cross-validation results before and after each dimension reduction are shown in Table 2. After the dimension reduction, enhancer recognition effect is obviously seen to be improved, but as the number of dimension reductions increases, performance is not getting better and better. Instead, the performance is the best when the dimension is reduced to 791 for the first time; therefore, we finally chose 791 dimensional features as the input of the classifier.

After adopting CNN, LSTM, and autoencoder for feature extraction, we failed to achieve ideal results. Since random forest is good at processing high-dimensional data and has strong anti-interference ability, we tried to use it for classification and finally achieved relatively ideal results. In the independent test, the model achieved an accuracy of 77.5% and MCC of 0.552.

### 3.2. Enhancer Classification

Since the model construction method of identifying enhancers is not ideal when applied to classifying enhancers, we considered introducing a new scheme. In terms of feature representation, *k*-mers are used to segment biological sequences in this paper, and after 3-mers, the 200 long strong and weak enhancer sequences will be converted to 198 words. For example, the sequence “TACATTCA” after 3-mers is divided into 6 words “TAC ACA CAT ATT TTC TCA”.

Then, the word2vec model is used to generate words into vectors to represent the relationships between words. word2vec relies on two training modes: continuous bag of words (CBOW) and skip-gram [39]. To achieve better results, we tried to use CBOW and skip-gram models with different parameters and compared their performance. In the experiment, parameters were adjusted from three aspects: the optimization method of the model training mechanism (negative sampling (NS)/hierarchical softmax (HS)), the minimum word frequency of the word vector (Min_count), and the maximum context distance of the word vector (Window). As shown in Table 3, when the CBOW model and HS, Min_count, and Window were set at 5 and 5, respectively, the ACC reached 67.57%, and the MCC was 0.3529, showing the best effect. Then, LSTM, which is a variant of RNN, is used for training. In this paper, the 5-fold cross-validation performance of LSTM, Bi-LSTM, and attention-based Bi-LSTM is compared. As shown in Table 4, the attention-based Bi-LSTM model performs better. An MCC of 0.4881 with an accuracy of 73.5% was achieved in an independent test.

A noteworthy problem is that this model and existing methods such as Enhancer-5Step and iEnhancer-ECNN have higher SN in the enhancer classification results, while SP is lower, at least 20% lower than SN. This shows that the model has a better ability to identify strong enhancers, while the ability to identify weak enhancers is weak. The potential reasons are roughly divided into two aspects: feature extraction and model construction. When the extracted features cannot distinguish weak enhancer samples that are similar to strong enhancer samples, it is identified as a strong enhancer. The second is model building. There are also great differences in the discriminative ability of different computational models for the same dataset. In this regard, we can try more feature extraction algorithms and classification algorithms in the future to improve this problem.

## 4. Material and Methods

### 4.1. Benchmark Dataset

In our study, a benchmark dataset was derived from Liu et al. [6]. This dataset is widely used in enhancer classification studies such as EnhancerPred and iEnhancer-EL. The dataset consists of 200 bp DNA sequences, and then in order to avoid redundancy, CD-HIT software [40] was used to delete pairwise sequences (sequences with similarity greater than 20%). Finally, we obtained the training set and independent set used by former researchers, in which the training set included 2,968 samples, and the ratio of nonenhancers, strong enhancers, and weak enhancers was 2 : 1 : 1. The independent test group is composed of 400 samples. Their number ratio is also 2 : 1 : 1.

### 4.2. Dinucleotide-Based Auto-Cross Covariance (DACC)

our research, we integrate the global sequence-order information into the model by using a feature extraction method based on DACC. It is formed by the combination of dinucleotide-based auto covariance (DAC) and dinucleotide-based cross covariance (DCC). In this combination, the DAC code calculates the correlation of dinucleotides along a lag distance between sequences with the same physical and chemical properties. The calculation form is as follows:

(1) $$\begin{array}{c} \text{DAC}\left(\varphi ,\text{lag}\right)=\overset{L-\text{lag}-1}{\underset{i=1}{\sum }}\left(\frac{\left(P_{\varphi }\left(R_{i}R_{i+1}\right)-\bar{P_{\varphi }}\right)\left(P_{\varphi }\left(R_{i+\text{lag}}R_{i+\text{lag}+1}\right)-\bar{P_{\varphi }}\right)}{L-\text{lag}-1}\right),\\ \bar{P_{\varphi }}=\overset{L-1}{\underset{i=1}{\sum }}P_{\varphi }\frac{R_{i}R_{i+1}}{L-1}, \end{array}$$

where *L* denotes the sequence length; *R*_*i*_ represents the nucleic acid residue located at the *i*^th^ position; *P*_*φ*_ is a physical and chemical property and *φ* is a physical and chemical property index; *P*_*φ*_(*R*_*i*_*R*_*i*+1_) on behalf of the position *i* dinucleotide *R*_*i*_*R*_*i*+1_ values correspond to the physical and chemical properties *P*_*φ*_; $\bar{P_{\varphi }}$ is the numerical mean value of dinucleotides corresponding to physicochemical properties in the whole DNA sequence.

For example, a DNA sequence with a length of 8 is “TACATTCA”, and the corresponding dinucleotide value under the “Shift” physicochemical property is shown in the Table 5. Then,

(2) $$\begin{array}{c} \bar{P_{\varphi }}=\frac{P_{\varphi }\left(\text{TA}\right)+P_{\varphi }\left(\text{AC}\right)+P_{\varphi }\left(\text{CA}\right)+P_{\varphi }\left(\text{AT}\right)+P_{\varphi }\left(\text{TT}\right)+P_{\varphi }\left(\text{TC}\right)+P_{\varphi }\left(\text{CA}\right)}{7}=\frac{-2.243+0.126-0.861-1.019+1.587+0.126-0.861}{7}\approx -0.449. \end{array}$$

**Figure 2:**
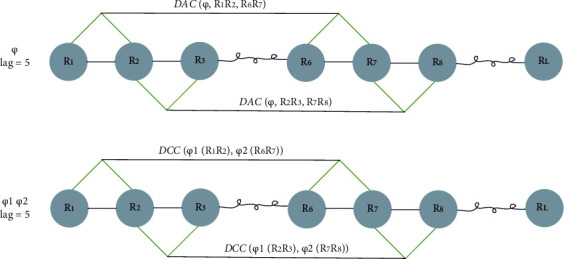
The process of generating DAC and DCC feature vectors of sequence “*R*_1_*R*_2_, ⋯, *R*_*L*_”.

When lag is 5 (as shown in Figure 2),

(3) $$\begin{array}{c} \text{DAC}\left(\varphi =\text{Shift},\text{lag}=5\right)=\frac{\left(P_{\varphi }\left(R_{1}R_{2}\right)-\bar{P_{\varphi }}\right)\left(\left(P_{\varphi }\left(R_{6}R_{7}\right)-\bar{P_{\varphi }}\right)+\left(P_{\varphi }\left(R_{2}R_{3}\right)-\bar{P_{\varphi }}\right)\right.\left(\left(P_{\varphi }\left(R_{7}R_{8}\right)-P_{\varphi }\right)\right.}{L-\text{lag}-1}=\frac{\left(P_{\varphi }\left(\text{TA}\right)-\bar{P_{\varphi }}\right)\left(P_{\varphi }\left(\text{TC}\right)-\bar{P_{\varphi }}\right)+\left(P_{\varphi }\left(\text{AC}\right)-\bar{P_{\varphi }}\right)\left(P_{\varphi }\left(\text{CA}\right)-\bar{P_{\varphi }}\right)}{L-\text{lag}-1}=\frac{\left(-2.243+0.449\right)\left(0.126+0.449\right)+\left(0.126+0.449\right)\left(-0.861+0.449\right)}{2}\approx -0.634. \end{array}$$

So, the DAC eigenvalue of the sequence “TACATTCA” is about -0.634 under the physicochemical property of “Shift” and when lag is 5.

The dimension of the feature vector is *N* × LAG after DAC, where *N* is the number of physicochemical properties and LAG is the maximum of lag (lag = 1, 2, ⋯, LAG). In this paper, LAG is 5.

DCC encoding was used to calculate the correlation of dinucleotides along a lag distance between sequences with different physical and chemical properties, and the calculation form was as follows:

(4) $$\begin{array}{c} \text{DCC}\left(\varphi_{1},\varphi_{2},\text{lag}\right)=\overset{L-\text{lag}-1}{\underset{i=1}{\sum }}\frac{\left(P_{\varphi_{1}}\left(R_{i}R_{i+1}\right)-\bar{P_{\varphi_{1}}}\right)\left(P_{\varphi_{2}}\left(R_{i+\text{lag}}R_{i+\text{lag}+1}\right)-\bar{P_{\varphi_{2}}}\right)}{\left(L-\text{lag}-1\right)}, \end{array}$$

where *L* denotes the sequence length; *P*_*φ*_1__ and  *P*_*φ*_2__ are the two different physicochemical properties; *P*_*φ*_*a*__(*R*_*i*_*R*_*i*+1_) on behalf of the position *i* dinucleotide *R*_*i*_*R*_*i*+1_ correspond to the physical and chemical properties of *P*_*φ*_*a*__, *a* = 1, 2; $\bar{P_{\varphi_{a}}}$ is the numerical mean value of dinucleotide corresponding to physicochemical properties of *P*_*φ*_*a*__ (*a* = 1, 2) in the whole DNA sequence.

Similarly, take the sequence “TACATTCA” as an example; *φ*_1_ is the physicochemical property of “Shift” and *φ*_2_ is the physicochemical property of “Slide”, and their corresponding dinucleotide values are shown in Table 5. It is known that $\bar{P_{\varphi_{1}}}=-0.449$; then,

(5) $$\begin{array}{c} \bar{P_{\varphi_{2}}}=\frac{P_{\varphi_{2}}\left(\text{TA}\right)+P_{\varphi_{2}}\left(\text{AC}\right)+P_{\varphi_{2}}\left(\text{CA}\right)+P_{\varphi_{2}}\left(\text{AT}\right)+P_{\varphi_{2}}\left(\text{TT}\right)+P_{\varphi_{2}}\left(\text{TC}\right)}{7}=\frac{-1.511+1.289-0.623+2.513+0.111-0.394-0.623}{7}\approx 0.109. \end{array}$$

When lag is 5 (as shown in Figure 2),

(6) $$\begin{array}{c} \text{DCC}\left(\varphi_{1}=\text{shift},\varphi_{2}=\text{slide},\text{lag}=5\right)=\frac{\left(P_{\varphi_{1}}\left(R_{1}R_{2}\right)-\bar{P_{\varphi_{1}}}\right)\left(P_{\varphi_{2}}\left(R_{6}R_{7}\right)-\bar{P_{\varphi_{2}}}\right)+\left(P_{\varphi_{1}}\left(R_{2}R_{3}\right)-\bar{P_{\varphi_{1}}}\right)\left(P_{\varphi_{2}}\left(R_{7}R_{8}\right)-\bar{P_{\varphi_{2}}}\right)}{\left(L-\text{lag}-1\right)}=\frac{\left(P_{\varphi_{1}}\left(\text{TA}\right)-\bar{P_{\varphi_{1}}}\right)\left(P_{\varphi_{2}}\left(\text{TC}\right)-\bar{P_{\varphi_{2}}}\right)+\left(P_{\varphi_{1}}\left(\text{AC}\right)-\bar{P_{\varphi_{1}}}\right)\left(P_{\varphi_{2}}\left(\text{CA}\right)-\bar{P_{\varphi_{2}}}\right)}{L-\text{lag}-1}=\frac{\left(-2.243+0.449\right)\left(-0.394-0.109\right)+\left(0.126+0.449\right)\left(-0.623-0.109\right)}{2}\approx 0.241. \end{array}$$

So, the DCC eigenvalue of the sequence “TACATTCA” is about 0.241 under the physicochemical property of “Shift” and “Slide” and when lag is 5.

The dimension of the feature vector is *N* × (*N* − 1) × LAG after DCC, where *N* is the number of physicochemical properties and LAG is the maximum of lag (lag = 1, 2, ⋯, LAG). In this paper, LAG is 5. Therefore, the final dimension of the eigenvector of DACC is *N* × *N* × LAG.

### 4.3. Density-Based Spatial Clustering of Applications with Noise (DBSCAN)

DBSCAN can find clusters of any shape and can identify noise, which can achieve a better clustering effect for physical and chemical property data [12, 41]. The clusters are customized according to the parameters, respectively, “eps” (*e*-neighborhood with data point as center and *eps* as radius) and “minPts” (minimum number of data points in *e*-neighborhood). The steps of the DBSCAN algorithm are listed in Supplementary Materials.

**Figure 3:**
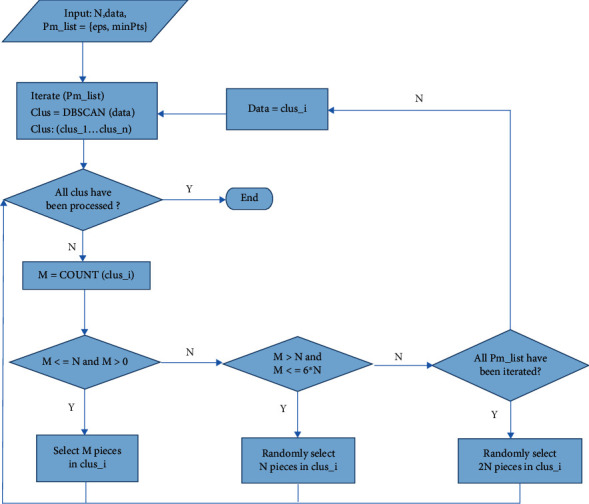
The process of screening physical and chemical properties by DBSCAN clustering.

**Figure 4:**
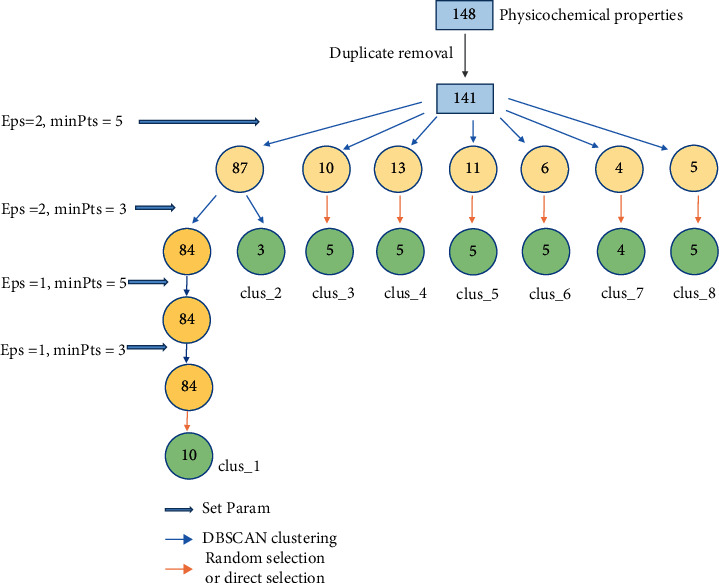
The specific situation after each clustering and the screening process of physical and chemical properties.

In this paper, DBSCAN was used to screen the physicochemical properties of dinucleotides. Our DBSCAN clustering process of data points is shown in Figure 3. First, to avoid overfitting, one of the equivalent physicochemical property indexes was randomly selected, and 141 kinds were obtained. Then, we input four sets of parameter values, which make the clustering algorithm increasingly strict. By observing the results of the first round of clustering in Figure 4, it can be found that except for the large number of data in the first cluster, the number of data in other clusters is between 4 and 13. In order to select an appropriate amount of physical and chemical properties from the clusters, we set the data threshold *N* as 5. According to the rules we made, we filter the clusters after each DBSCAN until all the clusters are processed. The number of data points obtained by each clustering is counted as *M*. The screening rule is that when *M* is between 0 and *N*, all physicochemical properties are selected. When *M* is between *N* and 6∗*N*, *N* pieces of data are randomly selected. When *M* is greater than 6∗*N*, the next set of parameters is used to recluster the cluster. If all parameters have been tried and *M* are still greater than 6∗*N*, 2∗*N* data will be randomly selected.

Figure 4 shows the number of clusters and the physicochemical properties number in each cluster after each cluster. Then, select the data in the cluster and a total of 47 dinucleotide physicochemical properties of 9 cluster types were finally obtained. The most representative physicochemical dinucleotide indexes were selected as much as possible, as detailed in Supplementary Materials.

### 4.4. Attention-Based Bi-LSTM

**Figure 5:**
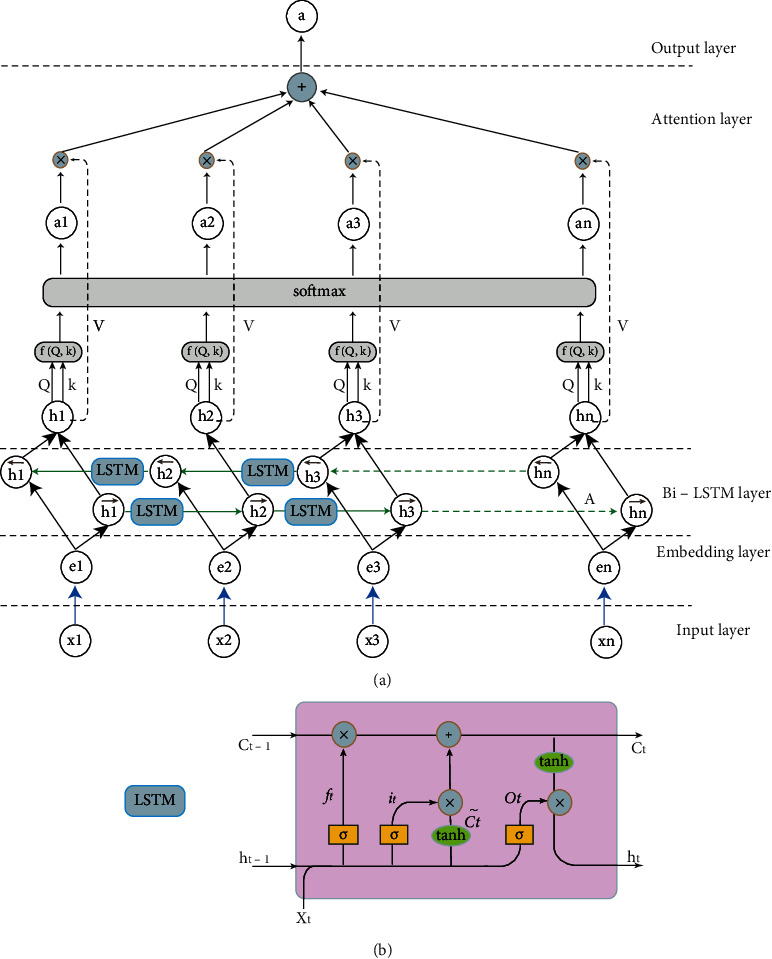
(a) The structure of attention-based Bi-LSTM. (b) The structure of LSTM in (a).

LSTM is a kind of time recurrent neural network that solves the long-term dependence problem of RNNs [42–45]. We can see the principal structure of LSTM in Figure 5(b), and its important components are the input gate, forgetting gate, and output gate. *C*_*t*_ is the cell state, which carries the memorized information and stores the information obtained through varied “gate” processing. *C*_*t*_ is similar to a kind of “long-term memory”, and *C*_*t*−1_ is the cell state of the previous stage. *h*_*t*_ is similar to a kind of “short-term memory”.

The first step in LSTM is to remove some information by working with the forgetting gate. The forgetting gate reads *h*_*t*−1_, *x*_*t*_ and passes through the sigmoid neural layer. The element value range of the output vector is 0~1, which represents the probability of information retention. The point-by-point multiplication operation updates the information to the cell state.

(7) $$\begin{array}{c} f_{t}=\text{sigmod}\left(W_{f}∙\left[h_{t-1},x_{t}\right]+b_{f}\right). \end{array}$$

The second step is to add new information from the input gate. The second step is divided into three steps: first, let the sigmoid layer of the input gate determine which parts of the information need to be updated, then let the tanh layer generate alternative updates, and finally, combine the two parts to add the information to the cell state.

(8) $$\begin{array}{c} i_{t}=\text{sigmoid}\left(W_{i}∙\left[h_{t-1},x_{t}\right]+b_{i}\right),\\ \tilde{C_{t}}=\tanh\left(W_{C}∙\left[h_{t-1},x_{t}\right]+b_{C}\right). \end{array}$$

The last step is to calculate and output the “short-term memory” state *h*_*t*_ by the output gate. First, let the sigmoid layer of the output gate decide the information part that needs to be updated; then, the tanh layer processes the cell state that has been updated and finally multiplies the two parts together to obtain *h*_*t*_.

(9) $$\begin{array}{c} o_{t}=\text{sigmoid}\left(W_{o}∙\left[h_{t-1},x_{t}\right]+b_{o}\right),\\ h_{t}=o_{t}∙\tanh\left(C_{t}\right). \end{array}$$

Therefore, the most special feature of LSTM is that it can forget unwanted information, add needed information, and obtain “short-term memory” according to “long-term memory” processing.

Bi-LSTM can better capture bidirectional semantic dependencies.  Figure 5(a)shows the Bi-LSTM structure in this article. After mapping, each word *x*_*i*_ obtains the word vector *e*_*i*_. After LSTM, the forward output is $\overset{⟶}{h_{i}}$, while the backward output is $\overset{⟵}{h_{i}}$. After Bi-LSTM, the vector obtained is $h_{i}=\left[\overset{⟶}{h_{i}}+\overset{⟵}{h_{i}}\right]$, where “+” represents the sum of corresponding elements. We can see in Figure 5(a) the Bi-LSTM layer.

Attention-based Bi-LSTM was first proposed by Zhou et al. in 2016 [46, 47]. Bi-LSTM with an attention mechanism avoids complicated feature engineering in traditional work. The attention mechanism allocates attention to each word in the process of learning the current information to make the model more focused on learning and thus improve learning efficiency [48]. The model has various variants, and self-attention [49] is adopted in this paper. Attention values can be calculated in three steps. Above all, we calculate the similarity between query (*Q*) and each key (*K*) by *f*(*Q*, *K*) to obtain weights. Then, the softmax function is used to normalize these weights. Finally, the weighted sum of the weights and the corresponding key value (*V*) is carried out to obtain the final attention value. In the self-attention model, query, key, and value are the same, that is, the input sentence sequence information *h*_*i*_ shown in Figure 5(a) which is the attention layer.

### 4.5. Model Evaluation

For evaluating and optimizing model performance, four evaluation indexes were used in this paper: ACC, SN, SP, representing accuracy, sensitivity, specificity, respectively, and MCC [38, 50–61]. Their mathematical formula is as follows:

(10) $$\begin{array}{c} \text{Accuracy }\left(\text{ACC}\right)=\frac{\text{TN}+\text{TP}}{\text{TN}+\text{TP}+\text{FN}+\text{FP}},\\ \text{Sensitivity }\left(\text{SN}\right)=\frac{\text{TP}}{\text{TP}+\text{FN}},\\ \text{Specificity }\left(\text{SP}\right)=\frac{\text{TN}}{\text{TN}+\text{FP}},\\ \text{MCC}=\frac{\text{TN}\times \text{TP}-\text{FN}\times \text{FP}}{\sqrt{\left(\text{TP}+\text{FP}\right)\left(\text{TN}+\text{FN}\right)\left(\text{TP}+\text{FN}\right)\left(\text{TN}+\text{FP}\right)}}, \end{array}$$

where TP,  TN,  FP, and FN represent the true positive, true negative, false positive, and false negative values, respectively.

## Figures and Tables

**Table 1:** The independent test performance comparison of this model with other models.

Method	ACC (%)	SN (%)	SP (%)	MCC
Enhancer identification				
EnhancerPred	74.00	73.50	74.50	0.4800
iEnhancer-2L	73.00	71.00	75.00	0.4604
iEnhancer-EL	74.75	71.00	78.50	0.4964
Enhancer-5Step	79.00	82.00	76.00	0.5800
Tan et al.	75.50	75.50	76.00	0.5100
iEnhancer-ECNN	76.90	78.50	75.20	0.5370
iEnhancer-CNN	77.50	78.25	79.00	0.5850
**Our method**	**77.25**	**77.30**	**77.20**	**0.5470**
Enhancer classification				
EnhancerPred	55.00	45.00	65.00	0.1021
iEnhancer-2L	60.50	47.00	74.00	0.2181
iEnhancer-EL	78.03	54.00	68.00	0.2222
Enhancer-5Step	63.50	74.00	53.00	0.2800
Tan et al.	68.49	83.15	45.61	0.3120
iEnhancer-ECNN	67.80	79.10	56.40	0.3680
iEnhancer-CNN	75.00	65.25	76.10	0.3232
**Our method**	**73.50**	**87.00**	**60.00**	**0.4881**

**Table 2:** In enhancer identification. The performance comparison of the 5-fold cross-validation before and after each feature dimensionality reduction.

Dimension	ACC (%)	MCC
11025	74.87	0.498
791	75.47	0.510
721	75.37	0.508
699	75.40	0.508

**Table 3:** The performance comparison of different parameters in the word2vec model in the enhancer classification in the 5-fold cross-validation.

	HS/NS	Min_count, Window	ACC (%)	MCC
CBOW	HS	3, 3	65.10	0.3123
		3, 5	66.22	0.3412
		5, 3	65.20	0.3119
		**5, 5**	**67.57**	**0.3529**
	NS	3, 3	61.82	0.2365
		3, 5	66.89	0.3381
		5, 3	63.76	0.2761
		5, 5	63.85	0.2908

Skip-gram	HS	3, 3	66.22	0.3258
		3, 5	65.54	0.3113
		5, 3	66.89	0.3379
		5, 5	65.20	0.3178
	NS	3, 3	66.44	0.3414
		3, 5	63.76	0.2768
		5, 3	64.09	0.2833
		5, 5	63.18	0.2754

**Table 4:** The performance comparison of LSTM, Bi-LSTM, and attention-based Bi-LSTM in the enhancer classification in the 5-fold cross-validation.

	ACC (%)	MCC
LSTM	64.21	0.2879
Bi-LSTM	61.41	0.2356
Attention-based Bi-LSTM	**67.57**	**0.3529**

**Table 5:** Under the physical and chemical properties of “Shift” and “Slide”, the corresponding dinucleotide values are involved in the sequence “TACATTCA”.

	TA	AC	CA	AT	TT	TC
Shift	-2.243	0.126	-0.861	-1.019	1.587	0.126
Slide	-1.511	1.289	-0.623	2.513	0.111	-0.394

## Data Availability

The data covered in this article can be found in Supplementary Materials.

## Supplementary Materials

Supplementary Materials The “S_47 physicochemical properties.txt” file is 47 of the 148 dinucleotides extracted after DBSCAN clustering of the physical and chemical properties. The “S_DBSCAN algorithm.docx” file is the DBSCAN algorithm framework. The “Training set.txt” file is the sequence samples in the training set. The “Independent testing set.txt” file is the sequence samples in the independent test set.
