# Supplementary material for: Identifying and Classifying Enhancers by Dinucleotide-Based Auto-Cross Covariance and Attention-Based Bi-LSTM
Source: Comput Math Methods Med. 2022 Apr 5;2022:7518779. doi: 10.1155/2022/7518779 (PMC9005296; doi:10.1155/2022/7518779)
Supplement: Supplementary Materials — The “S_47 physicochemical properties.txt” file is 47 of the 148 dinucleotides extracted after DBSCAN clustering of the physical and chemical properties. The “S_DBSCAN algorithm.docx” file is the DBSCAN algorithm framework. The “Training set.txt” file is the sequence samples in the training set. The “Independent testing set.txt” file is the sequence samples in the independent test set. [file 7518779.f1.zip › S_DBSCAN algorithm.docx]

| **Algorithm**: Algorithm of DBSCAN |
| --- |
| **Input:** Dataset D containing N objects: $D=\{\mathrm{dp}_{1},\mathrm{dp}_{2},\ldots,\mathrm{dp}_{N}\}$  $eps, \mathrm{minPts};$ |
| **Output:** $clus\_k (k = 1,2\ldots)$  **Initialize:** $v_{i}=0,\left( i=1,2,\ldots,N \right)$  1: Repeat |
| 2: randomly select a data point $\mathrm{dp}_{i}$ from D, D:=D\{$\mathrm{dp}_{i}$}  3: IF ($v_{i}=0$) // $\mathrm{dp}_{i}$ has not been visited  Compute the $N_{\mathrm{eps}}(i)$ //data points in its neighborhood eps (including itself)  Compute M // the number of data points in $N_{\mathrm{eps}}(i)$  IF $M<\mathrm{minPts}$, $v_{i} :=-1$ // temporarily mark $\mathrm{dp}_{i}$ as noise point  4: IF $M\geq\mathrm{minPts}$,  $v_{i} :=1$ //$\mathrm{dp}_{i}$ marked as the core point  $\mathrm{dp}_{i}\in clus\_k$ // $\mathrm{dp}_{i}$ grouped into the cluster clus_k  Let $N_{\mathrm{temp}}:=N_{\mathrm{eps}}(i)$  Repeat  randomly select a data point $\mathrm{dp}_{j}$from $N_{\mathrm{temp}}$, $N_{\mathrm{temp}}$:=$N_{\mathrm{temp}}$\{$\mathrm{dp}_{j}$}  IF $v_{j}\neq1,$let $\mathrm{dp}_{j}\in clus\_k$  IF $v_{j}=1$, $N_{\mathrm{temp}}:= N_{\mathrm{temp}}\cup N_{\mathrm{eps}}(j)$  Until $N_{\mathrm{temp}}$is Null  k := k + 1 // start the next clustering  Until D is Null |
|  |
